# A clinical trial comparing the effect of peer education and orientation program on the anxiety levels of pre-CABG surgery patients 

**Published:** 2015

**Authors:** R Esmaeili, Y Jannati, R Ghafari, JY Charati, HN Jelodar

**Affiliations:** * Orthopedic Research Center, Mazandaran University of Medical Sciences, Sari, Iran; ** Mazandaran University of Medical Sciences, Sari, Iran; *** Critical Care Nursing, Mazandaran University of Medical Sciences, Sari, Iran

**Keywords:** peer group education, employee orientation program, anxiety, coronary artery bypass grafting, clinical trial

## Abstract

**Background and Objectives:** One of the main treatment methods of coronary artery disease is coronary artery bypass graft (CABG) surgery. The anxiety level in patients undergoing this surgery is relatively very high. Thus, reducing anxiety in these patients is an important step toward wellness. This study aimed to compare the effects of peer education (PE) and orientation program (OP) on the anxiety levels of patients before CABG surgery.

**Material and Methods:**This randomized controlled trial was conducted in 2014 at the Mazandaran Heart Center on three groups of 50 persons each: PE, OP, and control (Cl). The anxiety levels of patients in each group were measured one day and one hour before the surgery. All groups received routine education. In addition, the PE group received PE and the OP group received OP. Two questionnaires were used to collect the demographics and the clinical data; and Spielberg state anxiety questionnaire was used to measure the anxiety level. Data from descriptive statistics, chi-square, ANOVA, ANCOVA, Bonferroni, and Fisher exact test were analyzed in SPSS v20 software.

**Findings:** The mean anxiety score before surgery was not significantly different in the three groups (P=0.955). However, after the intervention at 1 h before surgery, the mean anxiety level in the PE and OP group was lower than in the Cl group (P=0.000). However, the mean anxiety score between PE and OP groups showed no significant difference (P=0.051).

**Conclusion:** Both PE and OP group reduced the anxiety naturally developed in a patient before surgery. Although the influence of the PE group was greater in reducing anxiety, the use of this technique in clinical practices required further studies.

## Introduction

Cardiovascular diseases are the most common and significant cause of death worldwide [**1**]. The outbreak of these diseases is rapidly increasing in the Middle East, constituting about 40% of the total deaths [**2**]. The latest WHO report for Iran states that cardiovascular diseases are the first reason for deaths here, of which >45% of the deaths occur due to coronary artery diseases [**3**]. Despite the availability of facilities of medicinal treatment and invasive interventions, surgery requirement has significantly reduced in the present time; CABG surgery remains one of the major methods for treating coronary artery diseases [**1**]. One of the frequent problems of patients before the surgery is higher levels of anxiety in this case as compared to that in other surgeries, since heart is closely related to human life and death [**4**]. In a German research conducted in 2007, the pre- and post-CABG surgery anxiety level was 34–24.7%, respectively [**5**]. Lopez et al. [**6**] reported that the fear of surgery, getting into a strange setting, separation from family and its outcomes, feeling of helplessness, and the probability of death during surgery are the most critical reasons for anxiety in CABG patients before surgery. Increased anxiety results in raised heart beat, hypertension, the possibility of dysrhythmia, delayed wound healing [**7**], increased infection risk, electrolytes and fluid imbalance, altered sleep pattern [**8**], higher post-operation pain, postponed discharge [**9**], and, finally, lower patients’ satisfaction with treatment style and nursing care [**10**]. Generally, two different methods, medicinal and non-medicinal, are applied to lower pre-surgery anxiety of the patients. The non-medicinal ones have fewer side effects and risks as compared with the medicinal methods. These methods were not prescribed by the doctor. In addition, their implementation was easier, safer, and non-invasive [**11**].

One of the appropriate non-medicinal methods that can reduce the anxiety level is the provision of sufficient information and training about the disease, its control, and care, and follow-up program by the knowledgeable people involved in the same disease. In this training method, a plain, intimate, and secure setting is created, where the patients mutually share their experiences and feelings about the malady afflicting them. As a result, they utilize their peers’ fruitful and constructive experiences as a model, which promotes the disease treatment process and alleviates the associated symptoms [**12**]. The peer education (PE) group members could communicate better with their peers (patients) and encourage them to conduct themselves in suitable healthy behaviors, since they can share their weak and strong points as well as experiences at negligible or no cost [**13**]. Supporting the peers makes the patients more prepared mentally for the heart surgery and motivates them for a positive approach toward cardiac recovery [**14**].

On the other hand, since the fear for unknown as a result of lack of knowledge and unfamiliarity with the setting and the manner in which the cardiac surgery is performed, are the most initial and major reasons behind anxiety and worry [**15**], offering a program that familiarizes patients with the operating room, ICU, and the available devices in these wards seems like a significant approach toward reducing the patients’ pre-operation anxiety [**16**]. In an orientation program (OP), the information, cognitive and speech power, experiences and knowledge, and, consequently, their awareness about the events and phenomena in the setting increases [**17**]. Nowadays, in Iran, previous studies on training and familiarization of patients undergoing surgery have been conducted in the consulting rooms and pre-operation the familiarization of patients with the ICU and operating room is very infrequent. To raise self-care related knowledge, awareness, and skill via OP, allows the patient to get better adjusted with the problems of coronary artery bypass graft (CABG) surgery, such as anxiety [**18**].

Considering the exacerbated anxiety levels in patients about undergoing CABG surgery in comparison with other surgeries [**4**], no study has compared the effect of training by the PE and OP on pre-CABG surgery anxiety levels of patients. This question that arises regards which of the two methods, namely PE or OP, is more effective in lowering the patients’ anxiety levels in a research design ahead of CABG surgery.

## Method

This research is a clinical trial conducted with the aim of comparing the effect of training by PE and OP on the patients’ anxiety levels before CABG surgery. The study units were selected by a convenient sampling method. Then, the samples were assigned to either of the groups by a random block allocation. In the present study, a different block was selected every week, for example, the control group in the 1st week, the OP group in the 2nd week, and the PE group in the 3rd week. In order to prevent data contamination and to create a bias, the sampling process was started each week, depending on the study units discharged in the previous week, and, when no unit was discharged, sampling was not performed until it occurred. The sample size was determined with a confidence coefficient of 95% and a test power of 80%, 50 subjects being taken into account for each group. The inclusion criteria were include the patients undergoing CABG surgery for the first time, at least 18 years of age; fully conscious and knowledgeable regarding the time, place, and person; lacking physical and cognitive disorders; lacking medical education or related to it; no prior medical diagnosis of anxiety and depression; and not taking tranquilizer, anti-depressants, or anti-anxiety drugs at one month before surgery. The exclusion criteria were the unwillingness of patients to continue the participation in the study, and their condition worsening during the study period. The data collection tool consisted of three parts: personal traits, medical data, and Spielberg state anxiety questionnaire. To define the validity of the questionnaire’s first and second sections, face validity was employed, such that this questionnaire was presented to 10 people of the Mazandaran University of Medical Science faculty staff and modifications were made to the questionnaire post receiving their comments. The state of anxiety is the same instantaneous individual anxiety expressing the person’s current feeling or emotion at a time period like getting prepared for the surgery [**19**]. The Spielberg anxiety questionnaire had global validity and reliability. According to Mahram’s report for the mentioned test validity, the mean anxiety of normal and standard community in all age brackets was compared at 5% and 1%, to achieve a meaningful result, indicating the validity of anxiety measurement. The scientific reliability was also verified by α-Cronbach formula, which was 0.9452 in the normal community and 0.9418 in the standard community [**20**]. In addition, its reliability and validity in the Iranian society cardiac patients was confirmed via the study by Akbarzade et al.[**21**].

The questionnaire was made up of 20 multiple questions, with the options of “very little, little, a lot, and very much”. This questionnaire’s minimum score was 20 and the maximum was 80. In this research, the score 20–39 indicated mild anxiety, 40-59 indicated average anxiety, and 60-80 indicated intense anxiety [**22**].

After receiving the agreement from the research deputy of Mazandaran University of Medical Science and presenting the same to the Director of Mazandaran Heart Center, the researcher was referred to this center, and the list of patients undergoing CABG surgery during the past years was analyzed, seeking their peers. The inclusion criteria for peers covered the following ones: holding the least education level as a diploma had a successful CABG surgery at least a year before, having the right social relations, and having no anxiety or mild anxiety based on the Spielberg state anxiety questionnaire.

In the current study, in order to match the peers with the patients in terms of age and gender, two peers were selected to train the patients, of whom one was a 50-year-old man who trained male patients and a 45-year-old woman who trained the female patients. Then, the peers were trained by the researcher according to the CABG surgery candidate patients’ educational needs, based on the literature review, by holding an hour-long educational meeting. In the first meeting based on the study goals, the peers were trained regarding the concepts, importance, and advantages of PE training and anxiety lowering strategies such as taking a deep breath, listening to music, reciting prayers, and reading the Quran, post which they shared conveying their experiences. In the second meeting, peers were provided briefs on the post-CABG surgery care, physical activity level, diet, usual activity level, and treatment follow-up. In the third meeting, the other requirements of CABG candidate patients such as wound care, medicine supplementation, and diet, the required discussion, and training were addressed, the peers sharing their experiences in this field. Some gifts were given to the peers after the PE training meeting. The researcher’s PE training was provided in the form of lectures and question-answer sessions. One day before the surgery, the candidate patients were subjected to medical traits and Spielberg state questionnaires, prior to which a written informed consent was obtained from all patients. In this research, the patients with average (score: 40–59) or severe (score: 60–80) anxiety were included. The control patients only received the routine ward training, involving training by the physician, nurse, or via educational pamphlet. For the OP patients, in addition to the routine ward education, the researcher’s OP was conducted one day before surgery along with a 30-min excursion in the ICU and operating room in order to familiarize the patient with the ICU, the operating room, the staff, equipment, and devices existing in the wards. The researcher answered any question of the patients, and, finally, the CABG educational manual was offered to the candidate patients. Moreover, the PE group of patients received PE by the peers for one hour (training about the PE group importance and merits, anxiety reduction strategies, and pre-and post-CABG surgery cares) in the afternoon before the surgery, in addition to the routine ward training. Next, the patients’ anxiety was measured at one hour before the surgery. Finally, the data resulting from descriptive statistics, Chi-square, ANOVA, Covariance analysis, Bonferroni, and Fisher exact tests were analyzed by SPSS V.20. The significance level for all the tests was taken as 0.05. Ethical considerations in this research included describing the study goals and nature, acquiring a written informed consent from the patients, offering the research results to the study center officials and the patients’ families if requested, assuring the patients about maintaining confidentiality of their information and that they could leave the study at their will. 

**Findings **

Our results demonstrated that the mean age of the control, OP, and PE groups were 61.40 ± 7.92 years, 61.40 ± 7.50 years, and 63.84 ± 9.50 years. Most of the patients in these three groups were male, married, self-employed, under-diploma holders, or villagers. Chi-square statistical test revealed no meaningful difference among the three groups in terms of gender, marital status, education, and residency. In addition, no significant difference was observed in the job type and age by the Fisher exact test and ANOVA, respectively. Thus, the three groups showed homogenous results in terms of these variables (**Table 1**). The results of the medical features displayed that a majority of the study units in these three groups had a history of surgery and hospitalization. Chi-square test did not reveal any significant difference among the three groups in terms of medical features. In other words, the three groups showed homogenous results in terms of the tested variables (**Table 1**). Covariance analysis test revealed that the mean anxiety on the day before the surgery of the control, OP, and PE group patients were 44.32 ± 3.99, 44.58 ± 4.98, and 44.48 ± 3.91, respectively, which did not indicate any meaningful statistical difference (P=0.955) (**Table 2**). However, the patients’ mean anxiety scores for one day and one hour before the surgery of each group showed a significant statistical difference (P<0.05) (**Table 2**). Covariance analysis test results indicated that both the PE and OP methods reduced the patients’ anxiety levels in comparison with pre-intervention (P<0.05). Moreover, the results of the educational intervention effect analysis and comparison by PE and OP with that of the control group by using covariance analysis test, indicated that the anxiety score of the PE to control was lower (average 18.16; P<0.001), and the confidence interval (−15.963 to −20.354) and the anxiety score of the OP to control was lower (average 15.97; P<0.001), and confidence interval (−13.772 to −18.164).

The Bonferroni test results did not denote any significant statistical difference between the post-intervention mean anxiety score of the PE and OP groups (P=0.051) (**Table 1**).

**Table 1:** CABG candidate patients’ demographic and medical characteristics in the three groups of patients

Variable	Groups	Peer education		Orientation program		Control		Test result
		N	%	N	%	N	%	
Age	40–55	13	26	9	18	12	24	0.224
	56-70	26	52	35	70	31	62	
	71-80	11	22	6	12	7	14	
	Means	63.84		61.40		61.40		
	(SD)	9.50		7.50		7.92		
Gender	Man	32	64	34	68	28	56	0.45
	Woman	18	36	16	32	22	44	
Marital status	Married (having spouse)	43	86	48	96	42	84	0.128
	No spouse	7	14	2	4	8	16	
Education	Under diploma	40	80	35	70	44	88	0.084
	Diploma and higher	10	20	15	30	6	12	
Employment	Office worker	2	4	3	6	2	4	0.861
	Self-employment	20	40	20	40	22	44	
	Retired	13	26	10	20	6	12	
	Housewife	14	28	16	32	19	38	
	Jobless	1	2	1	2	1	2	
Residency	Urban	18	36	18	36	19	38	0.972
	Rural	32	64	32	64	31	62	
Surgery record	Yes	31	62	29	58	27	54	0.72
	No	19	38	21	42	23	46	
Hospitalization	Yes	44	88	36	72	37	74	0.141
	No	6	12	14	28	13	26	

**Table 2:** Comparing the mean and standard deviation of pre-and post-intervention CABG candidate patients in three groups of patients

Groups	Peer education	Orientation program	Control	P value
Pre-intervention Mean ± SD	44.48 ± 3.91	44.58 ± 4.98	44.32 ± 3.99	0.955
Post-intervention Mean ± SD	31.54 ± 3.36	33.78 ± 6.11	49.62 ± 5.09	0.001
P value	P<0.05	P<0.05	P<0.05	

**Fig. 1:**
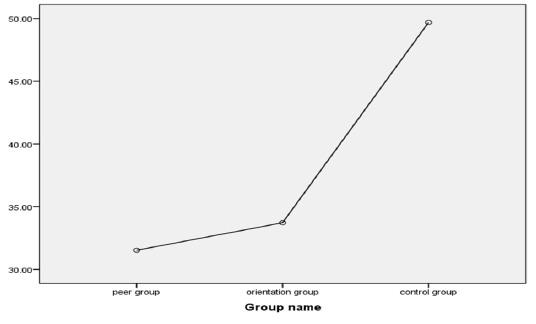
Post-intervention (1h before surgery) CABG candidate patients’ mean anxiety scores of the three groups of patients

## Discussion

Our study results demonstrated that both the educational methods by the PE and OP groups could effectively reduce the anxiety level of patients to undergo CABG surgery. The current research results suggested that the mean anxiety score of the three groups were not significantly different before the intervention and that the three groups showed homogenous results (P=0.955). Nasiriani et al. [**23**] indicated that the mean anxiety score of the test and the control groups did not have any meaningful statistical relationship the day before the surgery. These findings were consistent with those gained by few previous studies [**24**,**25**]. Our results implied that the use of PE to decrease CABG patients’ anxiety, as compared with the control group receiving no intervention, revealed a significant difference (P=0.000). Shamsizade et al. [**25**] researched about CABG surgery and found that PE is highly effective in lowering the anxiety and increasing self-efficacy in patients. In line with the present research, the findings obtained by Parent and Fortin [**24**] suggested that the PE group’s empathy-oriented support led to a decreased anxiety, increased self-efficacy, and self-report activity among the candidate patients for CABG surgery. Our results suggested that employing non-medicinal methods such as PE could be extremely effective in lowering the patients’ pre-surgery anxiety. According to these study findings, the mean anxiety score on one day before surgery (pre-intervention) in the OP group showed a significant difference compared with that of one hour before the operation (post-intervention) (P<0.05). The mean anxiety score at one hour before surgery in the control group did not decline, rather increased significantly (P>0.05). Nasiriani et al. [**23**] reported that the familiarization of the CABG surgery patients with the process of heart surgery reduced their anxiety score. The results of some previous studies were in concordance with the present ones [**17**,**26**]. Unlike in a previous study, Talaei et al. [**27**] showed in their survey that the familiarization method did not reduce the anxiety among patients. The authors conducted the OP, one day before the surgery and measured the patients’ anxiety then and again at half one hour before the surgery [**27**]. As Bruner said [**28**], the time when the patient was preparing for the operation was when she/ he experienced the highest level of anxiety and stress and it was difficult for a patient to control her/ his feelings at that time point. Thus, it was probable that factors such as not measuring post-surgery anxiety and re-comparing the groups with each other as well as measuring anxiety score at the time when the patient was preparing for the surgery could have affected the results. The result of this research did not indicate any meaningful statistical difference between the PE and OP groups toward lowering the patients’ anxiety level (P=0.051). In addition, in their study, Field et al. [**29**] discovered identical results, according to which, compared with the interpersonal psychotherapy, the PE supportive group did not reveal any significant difference in lowering the levels of anxiety and depression in pregnant women. Salavati et al. [**30**] compared the effect of personal education and PE on the quality of life of heart failure patients. They concluded that, although both the methods enhanced the life quality, the impact of PE group training was inconsistent as compared with the present research results [**30**]. In his research, Gharib [**31**] comparatively analyzed the effect of Benson relaxation and familiarization methods on the vital signs and anxiety of Endoscopic Retrograde Cholangiopancreatography (ERCP) candidate patients and demonstrated that the familiarization process was effective in reducing the patients’ anxiety level, which was incompatible with the results of the present study.

Our research limitations included the individual differences, and the psychological condition of the study units responding to the questions, which could have influenced the answering and the study units’ family, and socioeconomic problems, whose overall control was beyond the study potential.

## Conclusion

Our study results indicated the effect of education by the PE and OP groups on lowering the patients’ pre-operative CABG surgery anxiety. Both methods remarkably decreased the patients’ anxiety level. However, the impact of the two methods was equal, both being equally preferable. With respect to the findings of the present study, employing the two proposed non-medicinal, simple, effective, economical, and cost-effective approach was recommended to lower pre-surgery anxiety among the cardiac patients. We proposed to conduct such a study at a broader level and to prevent other patients from suffering of chronic diseases in the future.

**Acknowledgement **

This article is the result of the thesis in critical care nursing for a master degree that was registered in clinical trial site (code: IRCT2014102619677N1). Also, this project was conducted with the help of deputy of Research grant of Mazandaran University of Medical Science. We would like to thank the peers and patients who participated in this study.
